# Mechanistic insights into mutation in the proton-coupled folate transporter (SLC46A1) causing hereditary folate malabsorption

**DOI:** 10.1016/j.jbc.2025.108280

**Published:** 2025-02-07

**Authors:** Prithviraj Nandigrami, I. David Goldman, Andras Fiser

**Affiliations:** 1 Departments of Systems & Computational Biology, Albert Einstein College of Medicine, Bronx, New York, USA; 2 Department of Biochemistry, Albert Einstein College of Medicine, Bronx, New York, USA; 3 Departments of Medicine, Oncology and Molecular Pharmacology, Albert Einstein College of Medicine, Bronx, New York, USA

**Keywords:** PCFT, SLC46A1, hereditary folate malabsorption, molecular dynamics simulations, inactivating and compensatory mutations

## Abstract

Hereditary folate malabsorption (HFM) is a rare, autosomal recessive disorder characterized by impaired intestinal absorption and impaired transport of folates across the choroid plexus into cerebral spinal fluid due to inactivating mutations in the human proton-coupled folate transporter (hPCFT) gene, which encodes the proton-coupled folate transporter (PCFT) SLC46A1. Understanding the structural impact of these mutations is crucial for elucidating the mechanistic basis for PCFT function and the pathophysiology of HFM. Recently, the cryo-electron microscopic structural characterization of the *Gallus gallus* PCFT was obtained, which shares significant sequence identity with hPCFT. We conducted molecular dynamics simulations of hPCFT based on this structure, to explore structural changes induced by functionally defective disease-causing and other mutant proteins and mutations that restore function. Simulations revealed that the mutually mechanistic basis for the loss of function is partial loss of structural integrity of hPCFT primarily manifested in an enlarged and distorted pore accompanied by loss of long-range contacts, less stable, fluctuating inner helices with reduced solvent accessibility, and a marked loss of ordered secondary structures. These changes are reversed by the introduction of compensatory mutations. These findings provide novel insights into the structural and functional consequences of PCFT mutations associated with HFM and provide correlations with kinetic and biochemical properties of the mutant proteins.

Absorption of folates, a family of B9 vitamins, across the apical membrane of the proximal small intestine is mediated by the proton-coupled folate transporter (PCFT) SLC46A1, which is also required for transport of folates across the choroid plexus into the cerebrospinal fluid. Both processes are impaired when there are inactivating mutations in the human PCFT (hPCFT) gene causing the autosomal recessive disorder, hereditary folate malabsorption (HFM) (1, 2, 3). A variety of studies have identified PCFT residues associated with proton binding and coupling, folate and antifolate substrate binding, and residues that impact the rate of oscillation of the protein among its conformational states (4, 5). Studies focused on the kinetic and biochemical changes that occur with hPCFT mutations in HFM have provided insights into the structure-function of this transporter (1, 6). These studies were informed by the application of homology modeling that utilized sequentially remote template structures, such as the glycerol-3 phosphate transporter from *Escherichia coli* (7). More recently hPCFT was modeled on the bovine (inward-open) and rat (outward-open) Glut5 fructose transporter structures (8, 9) that share 88% sequence identity with each other but only 13% identity to hPCFT (10, 11). These past studies generated acceptable quality structural models for hPCFT, which provided insight into the general fold of the channel and residue positions affecting hPCFT function. However, these models were not sufficiently accurate for detailed studies of the structural changes associated with the mutant proteins. Recently, a cryo-electron microscopic structural characterization of the *Gallus gallus* PCFT (gPCFT) was obtained that shares 58% sequence identity with hPCFT (12). Hence, it became possible to better understand the structural and functional properties of hPCFT, and the basis for the defects associated with disease-causing mutations, by the application of molecular dynamics (MD) simulations (13, 14). Simulations performed in the current study provide insight into the mechanistic and structural changes induced by loss-of-function PCFT mutations and their reversal by mutually compensatory gain-of-function mutations.

A F392V mutation located in the endofacial region of the 11th transmembrane (TM) segment of hPCFT (Fig. 1) was identified (15) and characterized in a subject with HFM. The mutated protein is fully expressed, traffics to the cell membrane, but lacks function (11). Likewise, all other neutral, polar, and charged substitutions at this position result in a complete or marked loss of activity except for substitution with methionine for which function is nearly full preserved. Initial rate determinations for the V392H mutant, which retained sufficient transport activity to allow kinetic analyses, indicated that the loss of activity was due to a marked decrease in influx V_max_ without a change in influx K_m_. Studies based on the substituted-cysteine accessibility method (SCAM) were employed to further characterize, biochemically, the basis for functional changes associated with mutations at this site (10, 11). In this method, the accessibility of Cys-substituted residues is assessed for reaction with the aqueous-soluble, lipid-insoluble MTSEA-biotin alkylating reagent to define the aqueous translocation pathway and changes in accessibility of this pathway that may occur with alterations in the PCFT protein. These studies demonstrated that the F392V and other inactivating mutations at this site result in the loss of accessibility of Cys-substituted residues within the aqueous translocation pathway. Within the context of the alternating-access model, this suggested that the mutant proteins may be stabilized in the inward-open conformation (16). Earlier, a loss-of-function mutation (D109A) located in the cytoplasmic loop between the second and third transmembrane segments had also been shown to exhibit loss of accessibility on SCAM analysis (11). Interestingly, the addition of substitutions at two other positions, restored function of the F392V and, to a lesser extent, the D109A mutants: G305L in the eighth, and S196L in the fifth, TM segments, consistent with mutually compensatory mutation phenomena (17, 18). With this background of kinetic and biochemical data, a homology model of hPCFT based on the gPCFT structure, along with MD simulations, were employed to explore additional insights into the structural changes that occur with these mutant proteins. The results suggest that the basis for the loss of function of these mutant proteins is the general expansion and distortion of the pore. This disrupts critical contacts that impede the flow of folate substrate and reagents through the aqueous pathway, while apparently preserving sufficient biochemical integrity to allow trafficking of, and folate binding to, the protein. Upon the introduction of compensatory mutations these structural changes are reversed.

**Figure 1:**
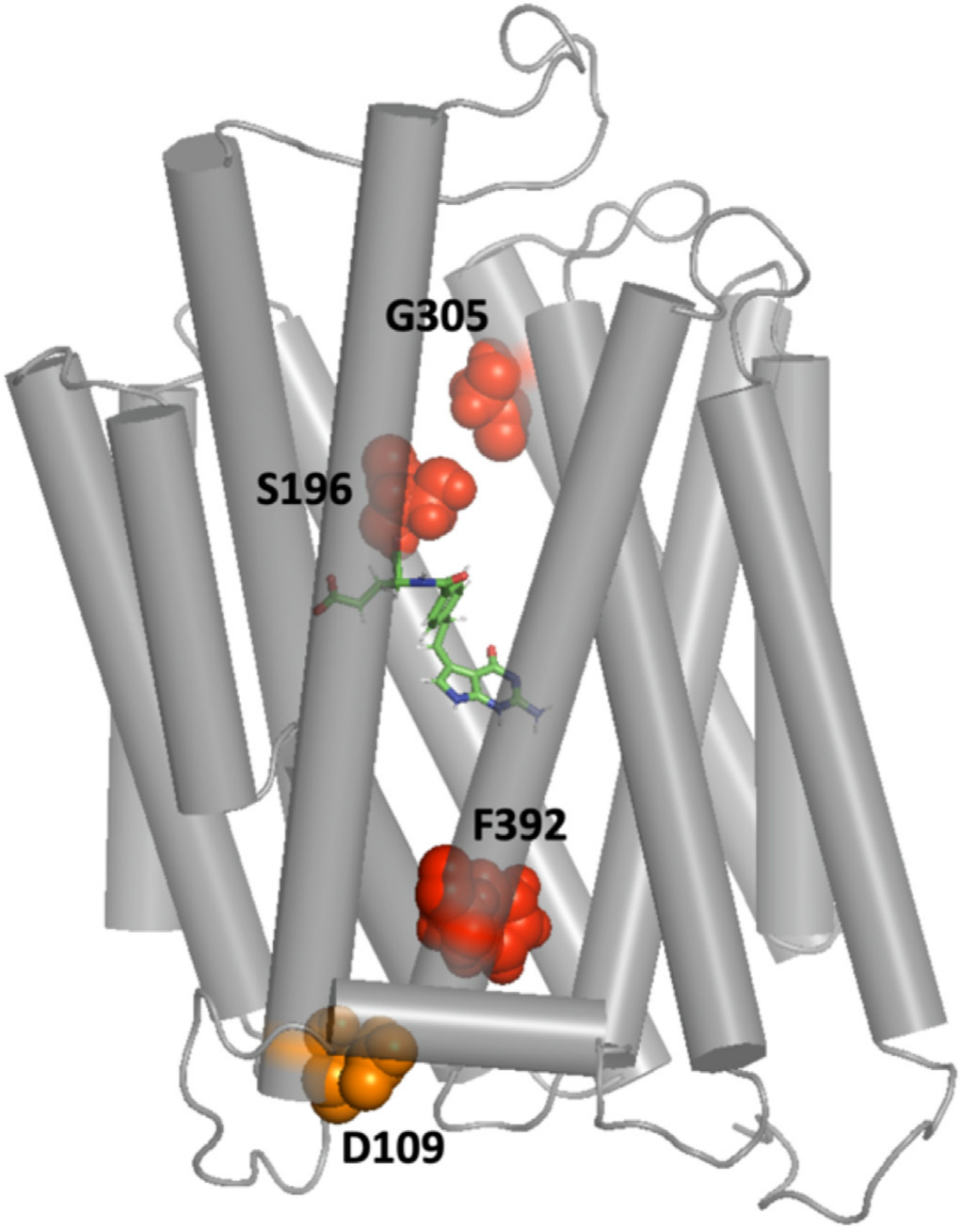
**hPCFT homology model based on the gPCFT structure (helices are shown in *gray cylindrical representation*) withresiduesF392, S196 and G305 (*red*), and D109 (*orange*)shownin *spheres*.** Pemetrexed is positioned in its binding site shown in *stickmodel*. gPCFT, *Gallus gallus* PCFT; hPCFT, human PCFT.

## Results

### Homology modeling and MD simulations

A broad spectrum of mutations at the F392 position of hPCFT result in markedly impaired function (11). The F392V mutation is inactivating; only F392M retains nearly full function. The F392D mutant is also inactive; minimal activity (20–30%) is retained with F392H or F392Y mutations. The loss of function with the F392V mutation is reversed by the addition of a S196L mutation and if the level of expression is considered, reversal appears to be near complete. The introduction of the G305L mutation to a F392V construct results in a lesser improvement of function. The S196L mutant alone is inactive, while the G305L mutant retains some activity (11).

To explore the molecular basis for these findings, homology models of hPCFT were constructed based on available cryo-EM structures of gPCFT (12), which share 58% sequence identity with hPCFT. Previous studies demonstrated that the accuracy of a homology model at this sequence identity level is comparable to a low resolution X-ray or a medium accuracy NMR solution structure (19). Homology models were generated for all single and double hPCFT mutations including F392V, S196L, F392D, F392D/G305L, and F392V/S196L. The gPCFT cryo-EM structure was solved with and without pemetrexed bound to the protein (12). Although the alternative structures only marginally differed (RMSD = 0.5 Å), both structures were used as starting points for simulations. These structures are assumed to be technical replicates since the difference between the two forms of gPCFT is comparable to the difference between the hPCFT homology model and gPCFT structure (all-atom RMSD = 0.4 Å). All homology models were tested for quality using three different methods, ProSA (20), DOPE (21) and ProQ3D (22). All quality benchmarking methods suggested that the homology models of hPCFT have a comparable quality to the gPCFT cryo-EM structure (Fig. S1, Tables S1, and S2).

**Figure 2:**
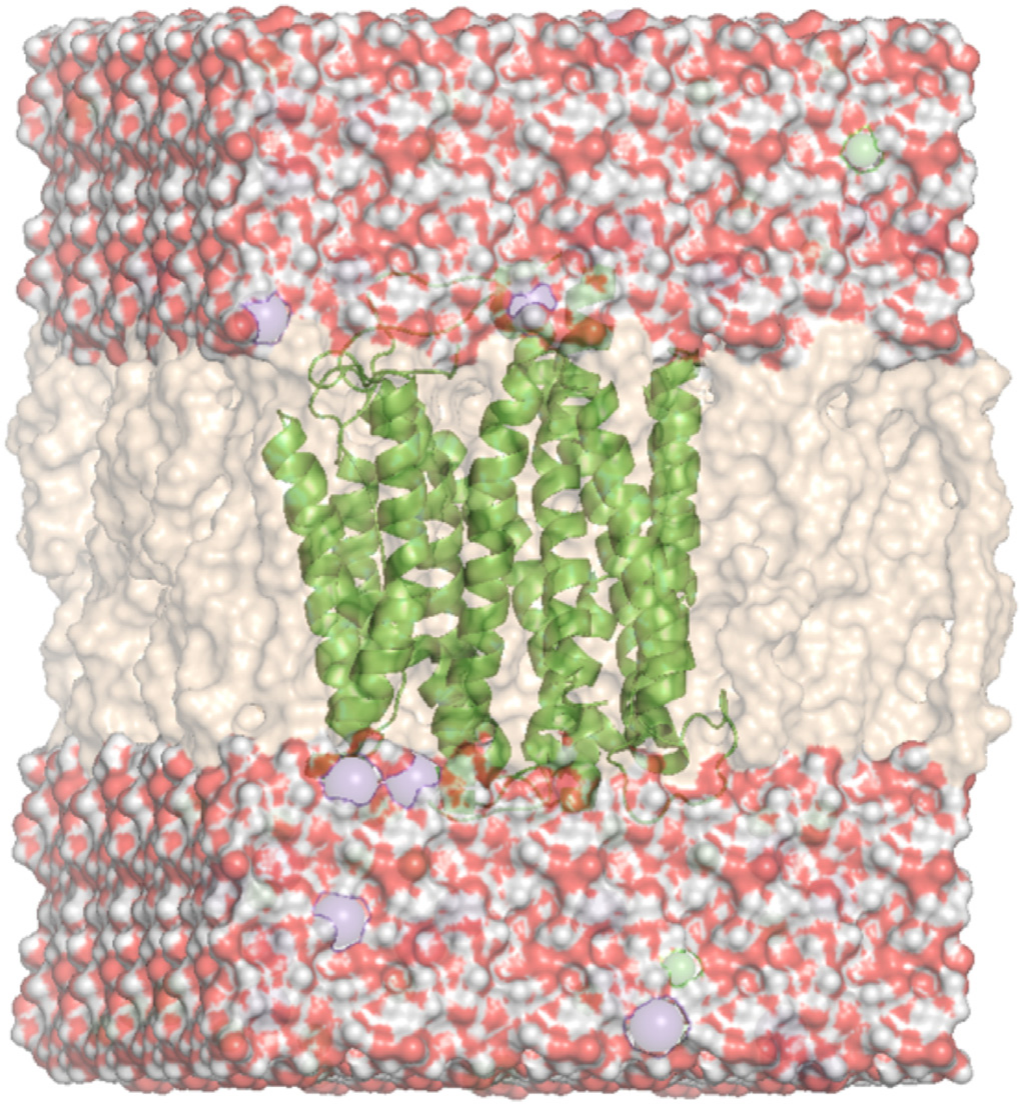
**Illustration of the molecular system simulated.** The hPCFT protein is shown in a *green ribbon model*, the lipid bilayer is shown in *tan* and the hydrogen and oxygen atoms of surrounding water molecules are shown in *white* and *red*, respectively. Ions calibrating the pH of the solvent are in various *color spheres: green* and *purple*. hPCFT, human PCFT.

All models were subjected to atomistic MD simulations embedded in a lipid bilayer surrounded by a water box and charge compensated by ions (Fig. 2). Approximately 1.5 μs simulations were run in triplicate for all models for reproducibility and generating sufficient statistics; 10,000 representative snapshots were extracted and analyzed from each simulated trajectory.

### Pore size changes induced with PCFT mutations

**Figure 3:**
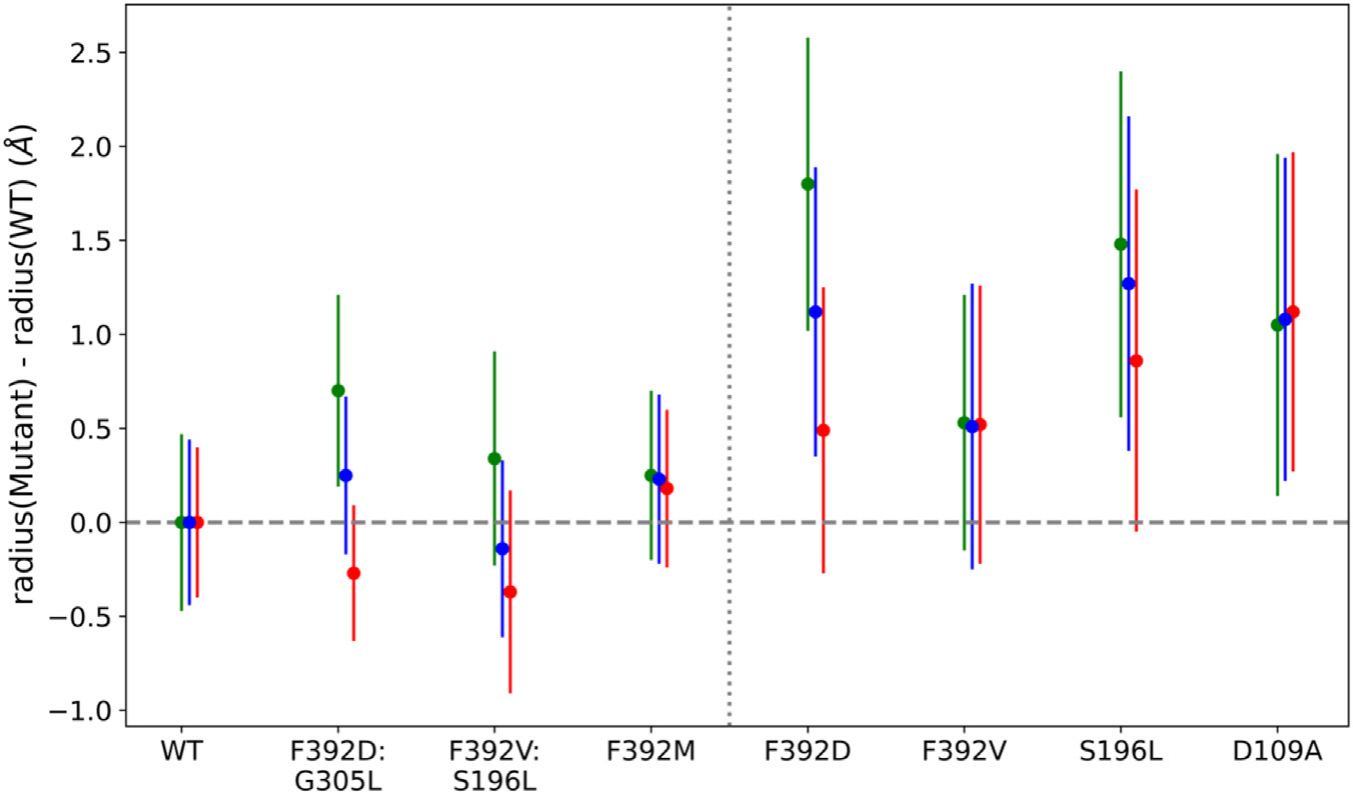
**Changes in the pore radius associated with hPCFT mutations.** The average pore radius of the various mutant hPCFTs is shown less the pore radius of the WT hPCFT reference. Starting models are based on pemetrexed-bound (*red*), pemetrexed-free (*green*) models, or the two combined (*blue*). *Vertical bars* indicate SDs calculated from six technical replicates from simulations starting with each starting model.

**Figure 4:**
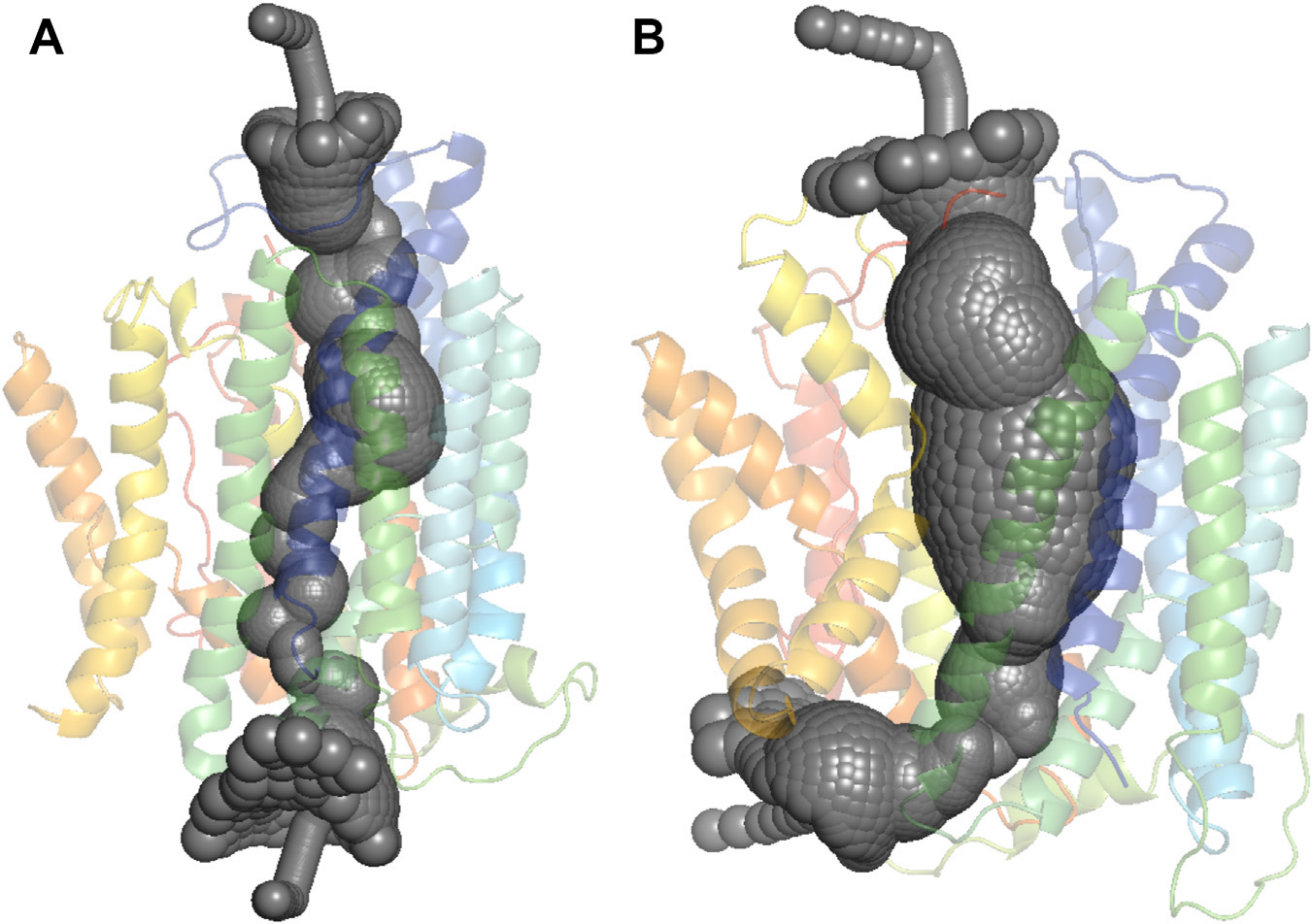
**Pore representation of PCFT.** Representative snapshots of hPCFT molecular models obtained from simulations are shown for (*A*) WT and (*B*) F392V mutants. The overall PCFT structure is shown in a *rainbow-colored ribbon model*. The calculated pore volume is filled in *dark gray spheres*. hPCFT, human PCFT; PCFT, proton-coupled folate transporter.

Among various structural features monitored, one of the most characteristic changes was observed with the pore size of the channel. The HOLE program (23) was used to calculate the average radius of the pore of all the hPCFT constructs to assess whether the size of the pore changed during the MD simulations. All single mutations that resulted in hPCFT dysfunction (F392V, S196L, F392D) resulted in distortion, and significant enlargement, of the pore radius as compared to WT PCFT (*p* values of differences = 0.0001, 0.0025, 0.0007, respectively) (Figs. 3 and 4). When compensatory mutations were incorporated in the models (F392V/S196L) (F392D/G305L), the pore radius relaxed back to a size comparable to the WT hPCFT pore size (*p* values = 0.242 and 0.335) (Fig. 3.). Any residue substitution at F392 resulted in a loss of function of PCFT, except for substitution with Met (11); hence as an additional test, we built and simulated a F392M hPCFT construct. In contrast to all other single substitutions at this position that impaired PCFT function and resulted in an enlarged pore, the radius of F392M hPCFT was not significantly different from the average WT hPCFT pore radius (*p* = 0.126). These results suggest that a functional aqueous pathway requires a well-packed pore, with properly positioned proton and substrate coordinating residues. Dysfunctional constructs apparently disrupt the proper residue coordination by relaxing the proper packing of the channel.

### Conformational dynamics

**Figure 5:**
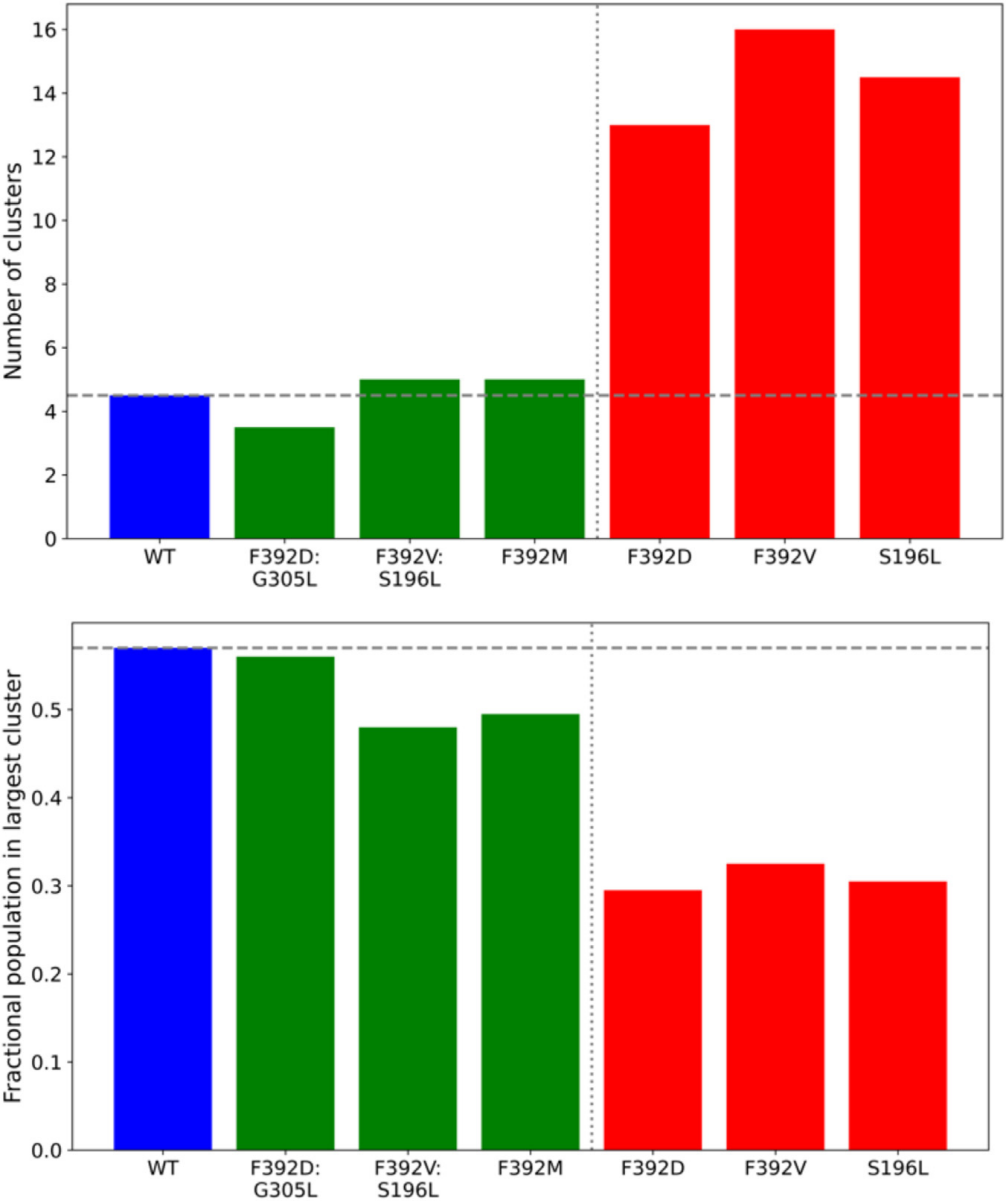
**RMS clustering of distinct conformations of the inner 8 TM helices of WT and mutant hPCFTs.***Upper panel*: Number of distinct conformational clusters. *Lower panel*: Fractional population in the largest cluster. Numbers of conformational clusters of WT and functional double mutants are shown in *blue* and *green colored bars*, respectively, while the dysfunctional single mutants are in *red*. hPCFT, human PCFT.

Two helices (TM4 and TM10), components of the core-forming 8 helices, along with TM8 are structurally broken in the midsection. Because of their flexibility, changes in these helices should be sensitive indicators of changes in structure. For instance, substitutions at the helix break of TM8 markedly increased the maximum rate of oscillation of the protein and, with relaxation of the protein, the affinity for folate substrates decreased (5). Changes in conformational clustering of TM4 and TM10 accompanying the various mutant hPCFTs were assessed over the course of MD simulations. The average number of distinct conformational clusters of these TMs were small (3.5–5) for the WT, F392M, and all the functional double mutants (F392V/S196L and F392D/G305L) as compared to functionally impaired hPCFT single mutants, which showed a 3 to 4 times higher diversity in the conformational clusters (Fig. 5). This suggests that the hPCFT structure becomes less well ordered when function is lost upon disruptive single mutations. The population of the single most dominant conformational cluster for WT hPCFT, the F392M mutant, and the functional compensated double mutants represents 48 to 56% of all conformations. In contrast, the most dominant cluster for single dysfunctional mutants contains only 29 to 33% of all conformations (Fig. 5), which suggests that dysfunctional mutant hPCFTs more frequently transition among a variety of alternative conformations.

### Impact of conformational changes on TM helices

**Figure 6:**
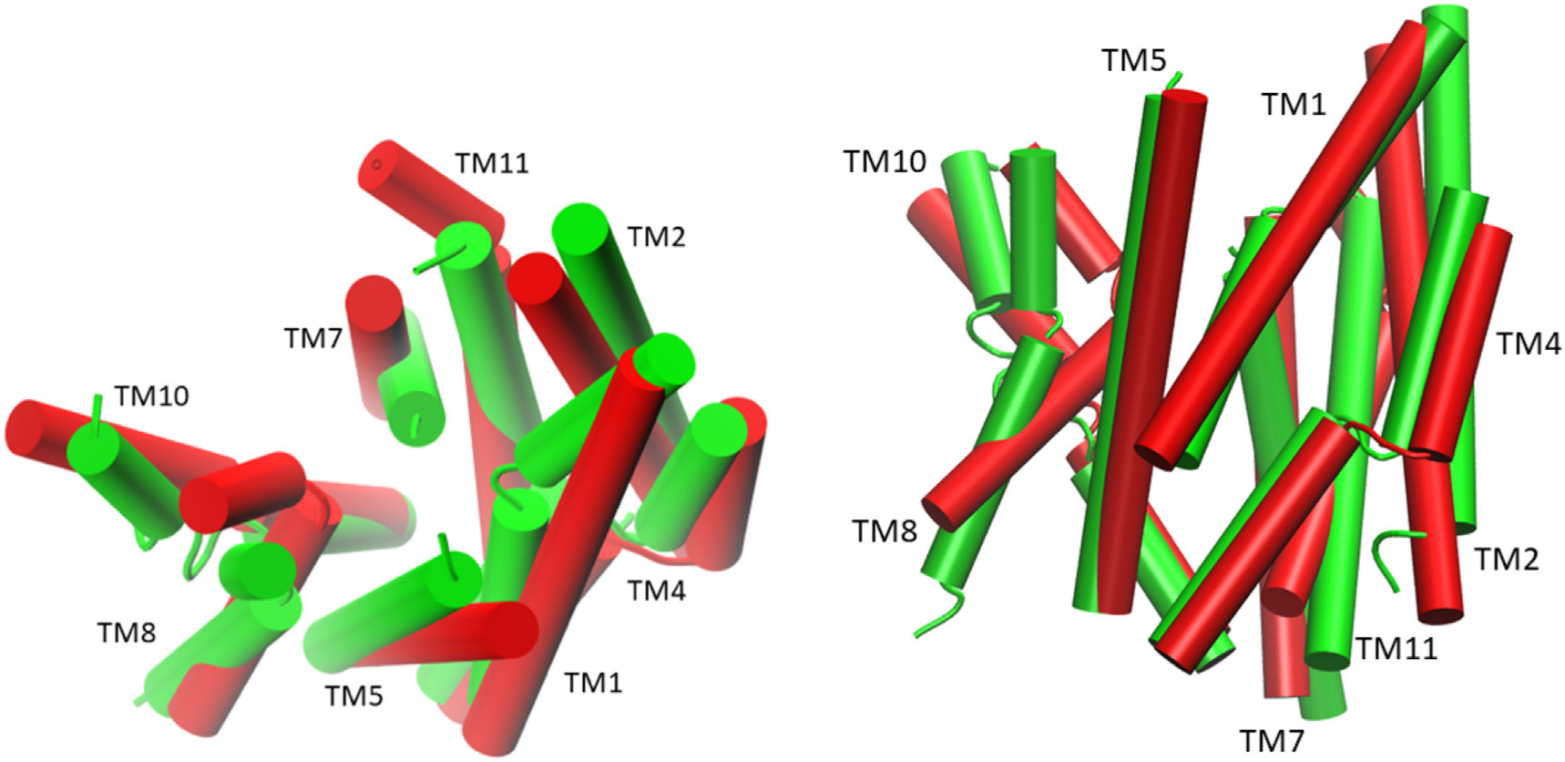
**Changes in the helix packing of PCFT upon mutation**. Representative molecular structures of hPCFT obtained from molecular simulations WT (*green*) and the F392V mutant (*red*) are superimposed. *Left:* a view into the aqueous pathway from the extracellular compartment. *Right:* a planar side view. Only the inner eight pore-forming TM helices are shown as indicated.

**Figure 7:**
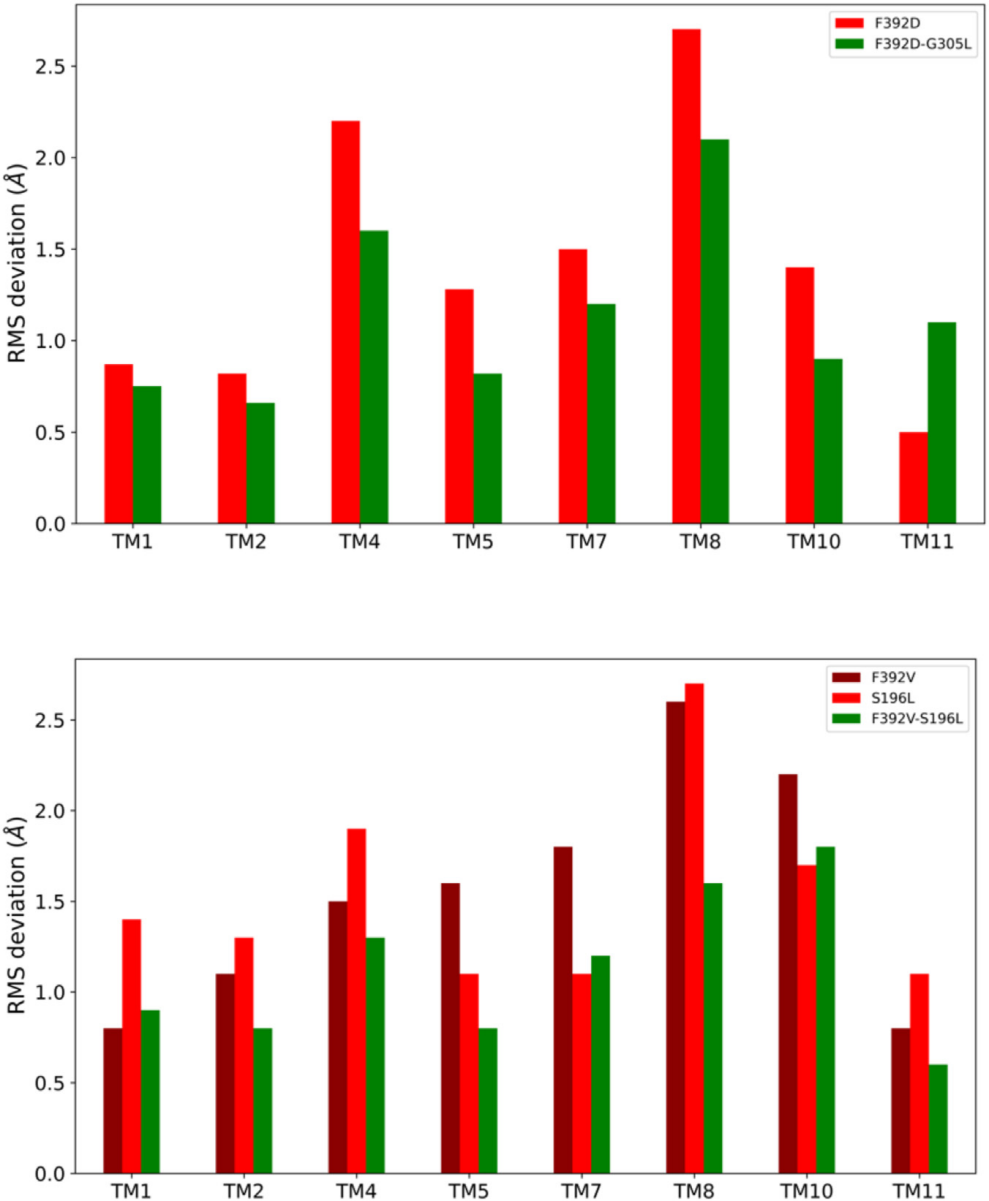
**Conformational changes in the inner 8 TM helices.** The similarity of each of the 8 TM helices are structurally compared (in units of RMSD) to the reference WT hPCFT for the two sets of mutants. *Upper panel*: F392D and F392D-G305L and (*lower panel*) F392V, S196L, and F392V-S196L mutants, respectively. hPCFT, human PCFT.

Analysis of conformational changes associated with the various hPCFT mutants was expanded to encompass all the inner 8 TM helices that form the PCFT aqueous pathway (TM1, TM2, TM4, TM5, TM7, TM8, TM10, and TM11). The total RMSD conformational change for the mutant hPCFTs as compared to WT hPCFT was analyzed as percentage contributions from individual TM helices. Values for a particular TM segment of the mutant hPCFTs close to zero indicate no significant conformational change, while larger values correspond to larger change. In both sets of mutants, TM7, TM8, and TM10 appear to undergo the largest structural changes despite the fact that two mutations that induce dysfunction are located in different helices (F392 is in TM11 and S196 is in TM5), only G305 is located in TM8 (Figs. 1 and 6). All the conformationally most affected helices are located in the C-terminal half of PCFT, which suggests an asymmetric pore dynamic (Fig. 7).

### Changes in solvent accessibility upon mutations

**Figure 8:**
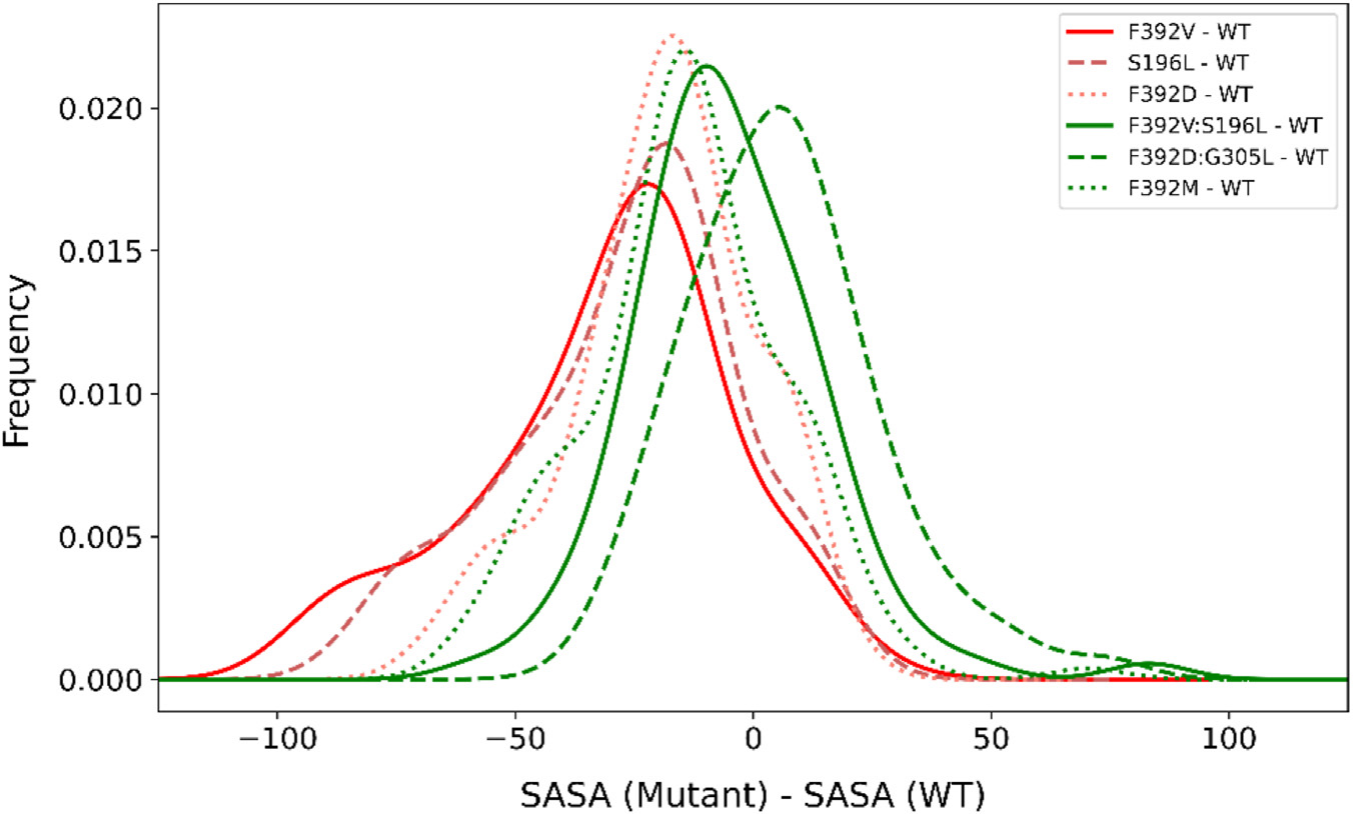
**Histogram of the difference in solvent accessibility for the eight inner TM helices between WT and mutant hPCFTs.** Dysfunctional mutant hPCFTs, (S196L, F392D, and F392V—*scattered, continuous, and dotted red lines*, respectively) have a reduced solvent accessibility compared to the functional WT, double mutant (F392D/G305L and F392V/S196L) or F392M hPCFT (*continuous, scattered, and dotted green lines*). hPCFT, human PCFT.

Previous studies using the SCAM suggested that the mutations at F392 decreased accessibility to the channel. To evaluate this further, changes in solvent-accessible surface area (SASA) were assessed among the various PCFT mutants (Fig. 8). There was a significant difference (*p* value < 0.0001) between the SASA of WT hPCFT and the dysfunctional single mutants (F392V, S196L, and F392D). In contrast, the double mutants, with restored function (F392V/S196L, F392D/G305L) and the only functional single mutant (F392M) were not significantly different in their SASA profile from WT hPCFT (*p* values = 0.3242, 0.7364, and 0.0063, respectively). Comparison of solvent accessibility between dysfunctional single mutants and WT hPCFT is consistent with a significant change in SASA for 236 residues; of these, 33 were increased and 203 were decreased in the dysfunctional single mutant F392D compared to the WT hPCFT. In contrast, the SASA change was significant in only 129 residues and well balanced (57 increase, 72 decrease) when a compensatory mutation was introduced (F392D/G305L). Similar SASA relative difference trends were observed for the dysfunctional F392V and S196L mutants as well (Fig. 8). For the F392V mutant, 221 residues changed in SASA value of which 58 increased and 163 decreased compared to the WT hPCFT, while for the S196L mutant, 228 residues showed a significant change in SASA of which 53 increased and 175 decreased compared to the WT hPCFT. When both mutations were introduced (F392V/S196L), only 152 residues showed a change in SASA of which 80 increased and 72 decreased in the double mutant compared to the WT hPCFT. Taken together, mutations that caused loss of hPCFT function resulted in an average decrease in solvent accessibility that was partially corrected when compensatory mutations were added, while the F392M mutant that retained function had overall unchanged solvent accessibility as compared to WT hPCFT (Fig. 8).

### Secondary structure content

**Figure 9:**
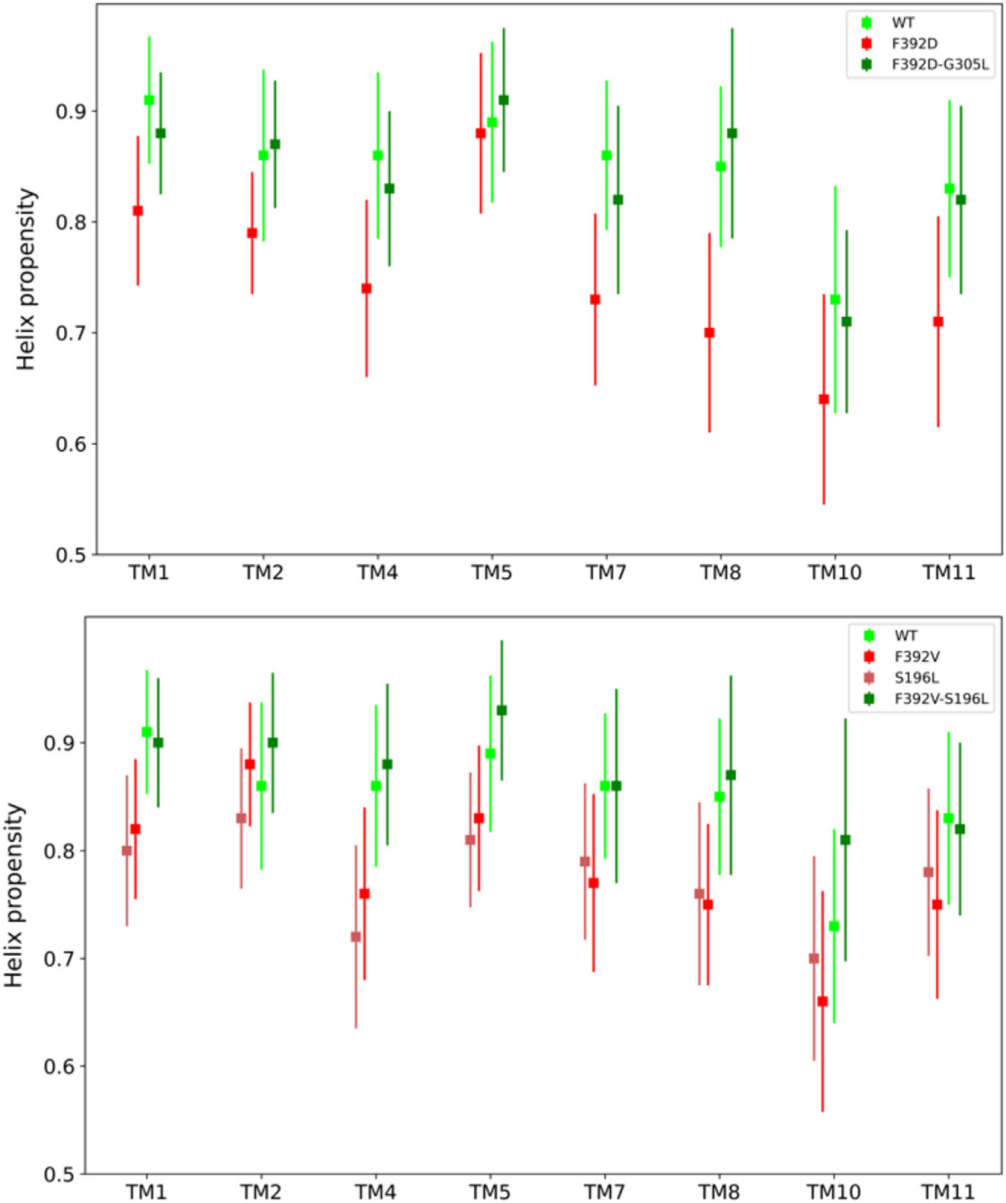
**Secondary structure (helix) propensity for each of the eight inner TM helices for WT and mutant (single and double) hPCFTs.** SDs are calculated from six independent simulations. hPCFT, human PCFT.

As indicated above, introduction of single mutations caused hPCFT dysfunction and a structural expansion of the pore, with several TM helices in the C-terminal section exhibiting the largest conformational shifts within the pore, and an accompanying change in solvent accessibility. Studies were undertaken to determine whether TM helices remain intact during these expansions shifting as rigid bodies or whether the TMs undergo intrinsic local conformational changes as well. The secondary structure content of these TM helices were calculated from 30,000 snapshots (10,000 snapshots from each of three replicates for a given hPCFT trajectory) of the corresponding MD simulation using the DSSP program that assigns secondary structures in three dimensional structures (24). All TM helices were found to have a significant reduction in their helical structure content (10–20%) except perhaps TM5 in the F392D/G305L construct and TM2 of the F392V mutant. This decreased helical integrity is recovered upon the introduction of compensatory mutations (Fig. 9).

### Residue contact analysis

**Figure 10:**
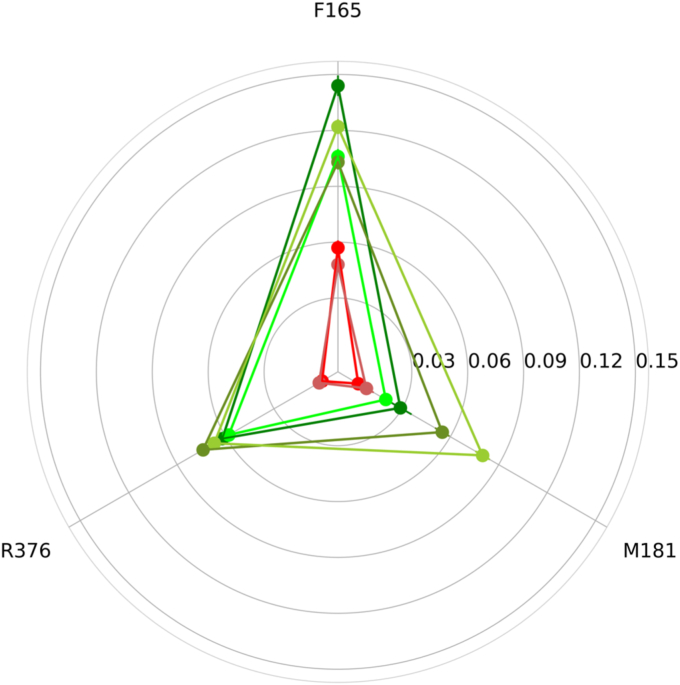
**Radar plot of contacts with the F392 residue.** The nodes indicate three long-range interacting residues (F165, M181, and R376). *Lines* and *nodes* that indicate functional proteins: WT PCFT (*green*), F392V/G305L (*dark green*), F392V/S196L (*olive*), and F392M (*lime*). *Lines* and *nodes* that indicate the loss-of-function mutants: F392V (*red*) and F392D (*brown*). PCFT, proton-coupled folate transporter.

Further studies with the INTERCAAT program (25) evaluated changes in the pattern of contacts of residue Phe392 with other residues with loss of function and, with the addition of compensatory mutations. Residues excluded ± six sequential positions in order to focus solely on long-range contacts. Three interacting residues were identified for residue F392: F165, M181, and R376. Interestingly, R376 was mutated in HFM patients and found to be critical to function (26). A radar plot (Fig. 10) indicates that these contacts are markedly disrupted with the loss-of-function mutants (F392V and F392D), while contacts are restored to WT levels when the compensatory mutations are added. Contact preferences were preserved for the F392M mutant, which retains full function and accessibility. These observations suggest that expansion and distortion of the pore with these mutations results in a loss of long-range contacts critical to proper packaging of the transporter.

### Pore radius analysis of D109A, a loss-of-function mutant located in a different region of the protein with similar SCAM findings as F392V

Studies were extended to determine whether an increase in pore radius was characteristic of another loss-of-function mutant, D109A, located between the second and third TM segments. This residue is irreplaceable and mutation results in a total loss of function. Like F392V, this mutation results in loss of accessibility of residues within the aqueous pathway based on SCAM analysis (27). Like F392V, the D109A mutation results in a 1.0 Å increase in the pore radius (Fig. 3).

## Discussion

Prior to the structural characterization of gPCFT, analysis of the accessibility of residues in the aqueous translocation pathway, “pore,” employed biochemical strategies with the SCAM. In this approach, cysteine-substituted residues are queried with an aqueous-soluble, lipid-insoluble, MTSEA-biotin reagent and residues alkylated (and thus assumed to be accessible and within the aqueous pathway) are subsequently pulled down with streptavidin and analyzed by Western blot (10, 28). With the solution of the gPCFT structure, it became possible to address the molecular mechanistic basis for functional changes in the transporter within the context of the known structure and to correlate MD simulation findings with the SCAM and kinetic results.

The current study focused on the functional changes at the F392 position based on the observation that the inactivating F392V mutation, identified in an individual with HFM, was associated with a loss of accessibility to MTSEA-biotin of cysteine-substituted residues within the aqueous translocation pathway as determined by a SCAM analysis. Interpreted within the context of the alternating-access transport model, the pattern of accessibility change suggested that the mutation stabilized the protein in the inward-open conformation. A unique finding was the identification of compensatory mutations; in particular, a mutually compensatory mutation, S196L, which alone also inactivated the protein but when cointroduced with F392V resulted in restoration of activity (11). The current study provides a structural perspective for the loss of function, a general expansion and distortion of the pore that is reversed with the introduction of the compensatory mutations. Accompanying the increase in pore radius was the loss of long-range residue contacts and a deterioration in organization of the F392V and other dysfunctional mutant proteins with the appearance of multiple alternative conformations, the loss of a dominant conformation seen in WT hPCFT, and a marked loss of integrity of the TM helices. These global and local structural changes due to inactivating mutations were accompanied by a reduced solvent accessibility pattern consistent with the SCAM results.

When the TM helices that define the aqueous pathway (TMs 1, 2, 4, 5, 7, 8, 10, and 11) were analyzed to determine which helices were most affected by the structural changes, TM7, TM8, and TM10 were identified. However, except for TM8, mutations that affected function were located in other TM segments. It is of interest that earlier studies based upon the generation of spontaneous disulfide bonds between Cys-substituted residues in the seventh and eighth TM segments indicated that the exofacial regions of these segments are in close apposition, suggesting that there is a deep exofacial aqueous-accessible cleft encompassing both helices (5, 10). Further, these three helices that undergo the largest conformational change are an important component of the C-terminal half of hPCFT suggesting an asymmetrical pore dynamic.

D109 was identified as another a residue that is essential for PCFT function; indeed, this residue is irreplaceable (29). D109 is located within the intracellular loop bounded by the second and third TM segments within a GXXXDXXGR(R/K) “motif A” (30). This motif at this location has been reported for a variety of members of bacterial SLC family members (30, 31, 32, 33). R113 (mutated in a subject with HFM) and G112, both components of motif A, were also shown to be critical for function (3, 27). Like F392V, the D109A mutant is expressed, traffics to the cell membrane, and exhibits impaired aqueous accessibility to MTSEA-biotin on SCAM analysis. Like F392V, MD simulation analysis of D109A indicated an increase in pore radius. Hence, increases in pore radius are observed with mutation of residues in the amino and carboxyl halves of the protein that are critical to function and the integrity of accessibility to the aqueous channel. It is of interest that residues in motif A as well as residues at the endofacial interface of the 11th TM segment, were shown to be required to achieve the outward-open conformation of the YajR transporter based upon mutational and structural analysis (30), observations consistent with the role these residues and regions play in PCFT function.

There is recent interest in applying MD simulations to the study of SLC transporters, particularly from the perspective of drug development (34). However, only a few studies that characterized disease-causing mutations such as SLC45A2 (35) and SLC24A5 (36) using structure modeling and MD simulations. None included SCAM and detailed kinetics analyses. In general, these mutations resulted in energetic instability and alterations in flexibility. There has been particular interest in disease-causing mutations involving the GLUT1 transporter (SLC2A1) that impair glucose transport across the blood-brain barrier into the central nervous system resulting in severe neurological and developmental defects (37). One mutant of GLUT1, R126C, shares the same kinetic basis for the loss of function as the F392 variants—a marked fall in influx V_max_ without a change in K_m_ (38). Similar to PCFT F392V, MD simulations of GLUT1 R126C indicated a marked decrease in protein stability but in neither case did this adversely impact on the expression of the protein and its trafficking to the cell membrane. Perhaps this can be explained, in part, by the observation that impaired stability in both cases was localized to specific segments of the proteins (39). It is of interest that despite the level of structural changes with alterations in solvent accessibility and the expected shifts in the location of binding residues, substrate affinities did not change for either the R126C-GLUT1 or F392V-PCFT mutants (38). Hence, in both cases the proteins appear to have tolerated substantial structural alterations, while preserving sufficient biochemical integrity to allow substrate binding, restricting the functional defect to impaired vectorial flow of folate substrates across the cell membrane.

Taken together, the data suggests that rather than stabilizing the protein in an inward-open conformation, impaired aqueous accessibility and impaired transport of folate substrates with these inactivating mutations is due to an enlarged and distorted pore accompanied by loss of long-range contacts, less stable, fluctuating inner helices with reduced solvent accessibility and a marked loss of ordered secondary structures. Clearly, these changes would also disrupt the orderly oscillation of the protein among its conformational states. The current study demonstrates the utility of MD simulations as an approach to better understand the structural basis for the functional changes that occur in inherited and other disorders caused by mutations in PCFT and other members of the SLC superfamily of transporters (40).

## Experimental procedures

### Homology model of hPCFT

Using chicken PCFT structures as template (pemetrexed-bound: Protein Data Bank (PDB) (41) identifier: (7BC7, and inhibitor free: PDB id: 7BC6), homology models of hPCFT were built in pemetrexed-bound and unbound conformations, respectively, utilizing MODELLER program (42). The two chicken PCFT templates (with and without inhibitor bound) are structurally highly similar, with an all-atom RMSD of <0.5 Å.

### MD simulations

All-atom, explicit solvent MD trajectories were generated for the following WT and hPCFT single and double mutant homology models: (a) WT hPCFT, (b) F392D mutant hPCFT, (c) F392D/G305L mutant hPCFT, (d) F392V mutant hPCFT, (e) S196L mutant hPCFT, and (f) F392V/S196L mutant hPCFT. For all cases, both a pemetrexed-bound and a pemetrexed-free gPCFT structure was used as template to generate homology models.

The Leap package (13, 14) implemented in AmberTools20 was utilized to parameterize and refine the starting model of hPCFT, which consists of modeling any missing atoms, terminal capping, and modeling disulfide bonds. The side chain rotamers were sampled at pH values at which the pemetrexed-bound and pemetrexed-free gPCFT models were solved (pH = 7.5 and 6.0, respectively). The *pdb4amber* tool (13) implemented in AmberTools20 was utilized to prepare the refined and optimized initial models to be used in subsequent MD simulations.

To prepare the lipid-embedded hPCFT models used as input in all simulations presented in this work, the 1-palmitoyl-2-oleolyl-phosphatidylcholine bilayer was modeled using the PACKMOL-Memgen tool by incorporating the refined input hPCFT model (as a PDB file) and 1-palmitoyl-2-oleolyl-phosphatidylcholine ratio in upper and lower leaflet as 1:1. The Oriented Patch Model coordinates were preoriented along *z*-axis for membrane building (43). All initial WT hPCFT and mutant hPCFT models were solvated using the TIP3P water model with appropriate ions added to achieve neutrality (44). A solvent buffer of 15 Å was placed on all sides of each membrane bound hPCFT starting model. A salt concentration of 0.15 M of KCl was added to the modeled water box. Periodic boundary conditions and particle mesh Ewald approximations for long-range interactions were implemented along with 9 Å cutoffs for electrostatic interactions. All MD simulations presented were performed with Amber20 using particle mesh Ewald CUDA implementation (pmemd.cuda) (45) and ff19SB (46) force field.

The minimization, equilibration, and production steps of all MD simulations were implemented and performed as following: (a) water and ions were minimized while other components were restrained with a positional restraint of 50.0 kcal/mol·Å^2^, (b) under constant volume, the system was slowly heated up from 100 K to 303.15 K over 20 ns of simulation time, (c) the system was kept at 303.15 K over 20 ns of simulation time at constant pressure, which allows the box density to relax, (d) keeping the system at 303.15 K at constant pressure, 10 ns of simulation was performed with a lower restraint of 10.0 kcal/mol·Å^2^, (e) minimization run was performed by applying restraint of 10.0 kcal/mol·Å^2^ on only the backbone atoms, (f) the system was relaxed over 10 ns of simulation time under constant pressure conditions with backbone atoms restrained with a coefficient of 10.0 kcal/mol·Å^2^, (g) the system was relaxed over 20 ns of simulation time under constant pressure conditions by lowering the restraint on the backbone atoms to 1.0 kcal/mol·Å^2^, and (h) the system was further relaxed over 20 ns of simulation time under constant pressure conditions by lowering the restraint on the backbone atoms to 0.1 kcal/mol·Å^2^. Following this minimization and relaxation protocol, production simulations with no restraints were run in the NPT ensemble at 1.0 bar and 303.15 K with an integration time step of 2.0 fs for a simulation length of at least 1.5 μs. The Langevin dynamics collision frequency was set to 1 ps^−1^ in production simulations. A unique random seed determined by the system clock was used for each Langevin dynamics simulation. Simulated trajectory frames containing solute and solvent atoms were collected at 100 ps intervals for subsequent analysis. Frames from the simulated trajectories were processed by scripts written using the CPPTRAJ (47) module in Amber20.

### Determining hPCFT core helices and their average fluctuation

Initial global fluctuation analysis of hPCFT MD trajectories (each hPCFT consists of 12 TM helices) suggested that the eight inner TM helices directly exposed to the pore would be the most insightful for structural comparison between different hPCFT mutants. These TM helices include TM1, TM2, TM4, TM5, TM7, TM8, TM10, and TM11. For each of the eight inner TM helices, RMSD in reference to the pemetrexed-bound and pemetrexed-free starting structures were calculated by averaging over ∼30,000 snapshots from three independent MD runs for each hPCFT model. RMSD of each of the inner eight TM helices measures overall similarity to the two reference structures.

### Average pore radius estimation of hPCFT channel

Average hPCFT pore radius fluctuations during MD simulations was calculated using the HOLE program (23), which measures average radius changes along X, Y, and Z directions for each residue position in the hPCFT channel.

### Clustering of structural ensembles of TM4 and TM10 during simulated trajectories

To gain insight into the dominant conformational modes of TM4 and TM10, a density-based conformational clustering approach was utilized with a threshold value of 1.5 Å (48).

### SASA over MD trajectories

The NACCESS (49) program (http://www.bioinf.manchester.ac.uk/naccess/) was used to calculate SASA for each residue of each hPCFT system simulated by averaging computed SASA values for each residue over three independent MD runs.

### Residue contact analysis

Residue contacts for F392 were determined with the INTERCAAT program (25). Ten thousand snapshots (30,000 snapshots in total for three replicates of each system) were used from the MD simulations, and for each structural snapshot, we calculated all the intramolecular contacts within 6.5 A (the default INTERCAAT setting). Contacts with residues within ±6 sequential positions were excluded to avoid counting of trivial short-range contacts.

## Data availability

The article contains all data described within the text and in Supplementary Materials.

## Supporting information

This article contains supporting information.

## Conflict of interest

The authors declare that they have no conflicts of interest with the contents of this article.
