# Supplementary material for: Mechanistic insights into mutation in the proton-coupled folate transporter (SLC46A1) causing hereditary folate malabsorption
Source: J Biol Chem. 2025 Feb 7;301(3):108280. doi: 10.1016/j.jbc.2025.108280 (PMC11929075; doi:10.1016/j.jbc.2025.108280)
Supplement: Supporting information [file mmc1.docx]

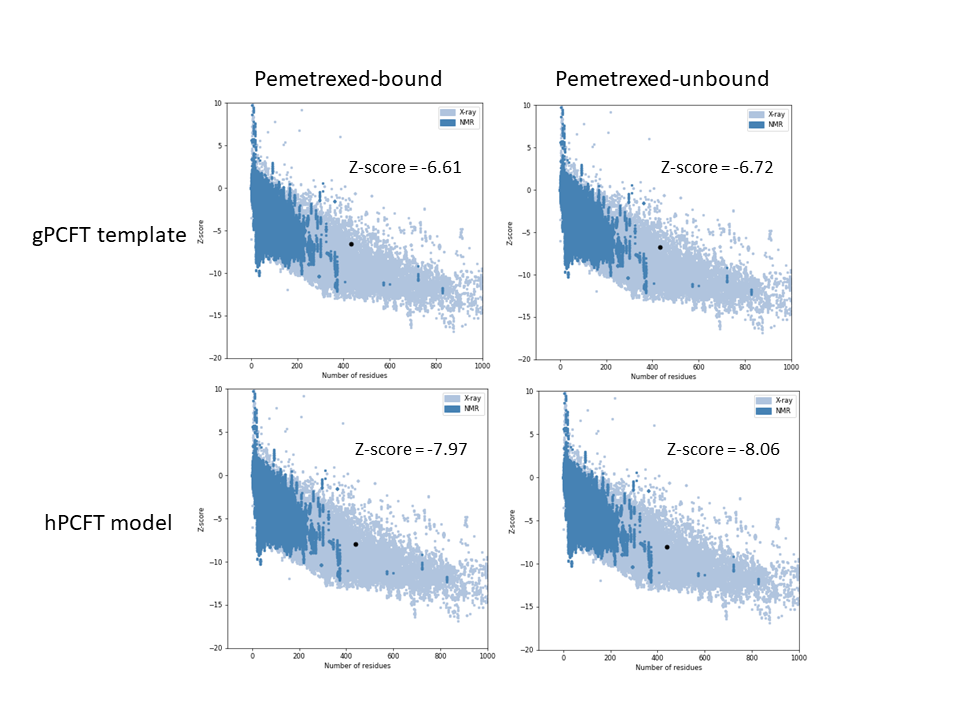


Figure S1: ProSA z-score evaluation and comparison of gPCFT templates and hPCFT models. Light and dark blue dots shows the distribution of z-score of X-ray and NMR structures of all chains in PDB database, respectively. The Z-score of pemetrexed-bound and pemetrexed-unbound gPCFT templates and hPCFT models are shown in black circle in each plot.


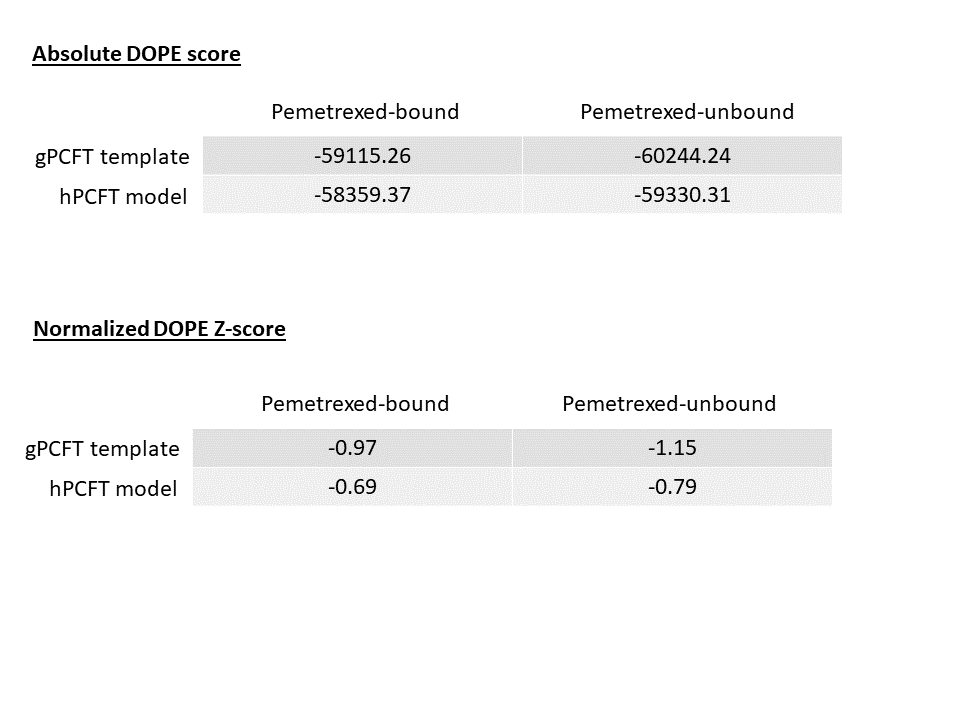


Table S1: Absolute DOPE score (top) and normalized DOPE Z-score (bottom) comparing of gPCFT template and hPCFT models.


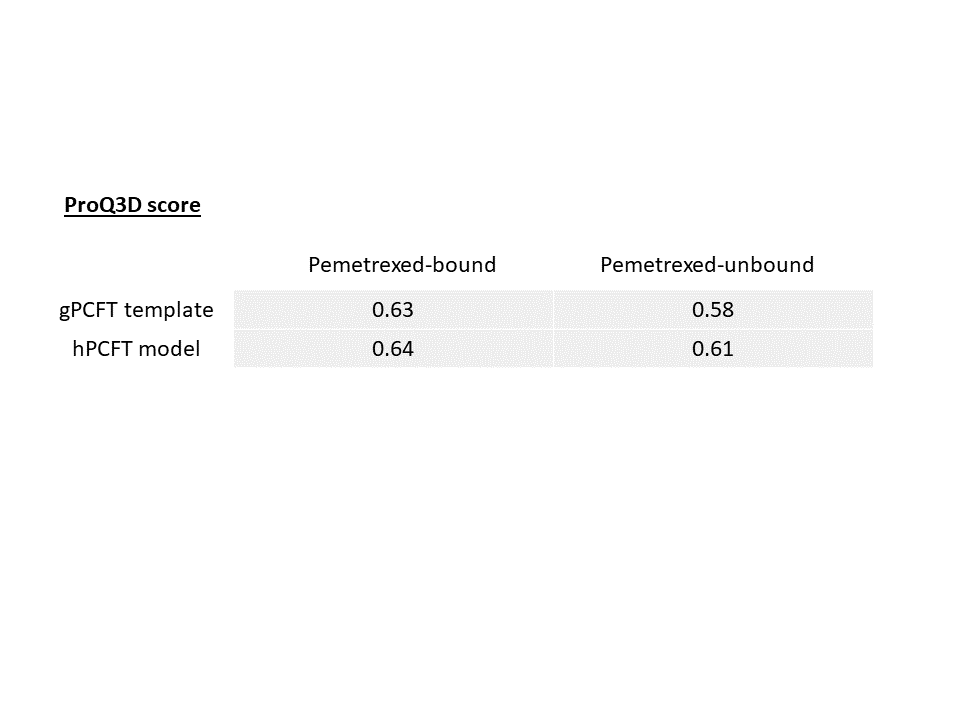


Table S2: ProQ3D score comparison of gPCFT template and hPCFT models.
